# Phytochemical Profile and Biological Activity of Endemic *Sideritis sipylea* Boiss. in North Aegean Greek Islands

**DOI:** 10.3390/molecules25092022

**Published:** 2020-04-26

**Authors:** Evangelos Axiotis, Eleftherios A. Petrakis, Maria Halabalaki, Sofia Mitakou

**Affiliations:** Department of Pharmacognosy and Natural Products Chemistry, Faculty of Pharmacy, National and Kapodistrian University of Athens, Panepistimiopolis Zografou, 15771 Athens, Greece

**Keywords:** *Sideritis sipylea* Boiss., phytochemical profile, total phenolic content, tyrosinase, elastase, antioxidant activity, ultrasound-assisted extraction (UAE), supercritical fluid extraction (SFE), UPLC-HRMS, GC-MS

## Abstract

*Sideritis sipylea* Boiss. is an endemic plant of the Mediterranean basin that is distributed in the Greek islands of the North Aegean Sea, i.e., Lesvos, Chios, Samos, and Ikaria, and in the West and Middle peninsula of Turkey. It is considered an endangered species because of its uncontrolled collection from its original habitat. Although the antioxidant, anti-inflammatory and antimicrobial properties have been previously reported, the total chemical profile has not yet been explored. In this context, the chemical profiles of the water/methanol (HA), methanol (ME), and ethyl acetate (EtOAc) extracts were analyzed using ultra-performance liquid chromatography coupled with high-resolution mass spectrometry (UPLC-HRMS). In parallel, analysis by gas chromatography-mass spectrometry (GC-MS) was employed for the dichloromethane extract (DCM) as well as for the essential oil (EO) and the extract obtained by supercritical fluid extraction (SFE). Furthermore, the total phenolic content (TPC) along with the in vitro tyrosinase and elastase enzyme inhibitory activity of different extracts was evaluated, towards the discovery of new active agents for cosmetic formulations. These activities are in accordance with its well-known antioxidant and anti-inflammatory properties, confirming the importance of ethnopharmacological references for *S. sipylea* in Greece and Turkey.

## 1. Introduction

The genus *Sideritis* belongs to the Lamiaceae family and consists of more than 150 species worldwide [1]. In countries of the Mediterranean, and especially in Greece, Italy, Spain, and Turkey, it is divided into 320 subspecies [2]. In Greece, the genus comprises eight species and seven subspecies with reference to the Mountain Flora of Greece [3]. A key characteristic of this genus is the classification difficulty because of the strong tendency of its species to hybridize and thus changing their chemical composition [4]. The phytochemical profile of *Sideritis* species has been extensively analyzed and thus the chemotaxonomic markers’ constituents of the genus, such as terpenoids, sterols, coumarins, flavonoid aglycones, and glycosides, are well known [4,5].

Many species of the *Sideritis* genus, such as *S. scardica*, *S. clandestina*, *S. syriaca*, *S. raeseri*, along with the endemic species *S. euboea* and *S. sipylea*, are commonly known as “mountain tea” or “ironwort” and have been widely used in traditional medicine, mainly as a decoction derived from the aerial parts. Numerous in vitro studies have previously shown that extracts from the aerial parts of *Sideritis* spp. demonstrate anti-inflammatory, antimicrobial, antifeedant, antihyperglycemic, antiulcerative, gastroprotective, spasmolytic, and bone-remodeling properties [6,7,8,9,10,11,12,13]. The aqueous extracts and volatile fractions from several species, including *S. sipylea*, have also demonstrated activities related to the Central Nervous System (CNS), acting as GABA_A_ receptor modulators [14,15]. *S. scardica* has shown potential for the treatment of neurological disorders and neurodegenerative diseases [15], while together with *S. galatica*, *S. lycia*, and *S. stricta* have been evaluated in vitro against Alzheimer and Parkinson disease [15,16,17].

Numerous secondary metabolites, with a strong antioxidant capacity, such as phenolic acids, flavonoids, and kaurene diterpenes, have been isolated from different extracts of several *Sideritis* species [2,11]. Moreover, flavonoids, such as xanthomicrol, have demonstrated a selective cyclooxygenase activity and therefore an anti-inflammatory activity. A strong structural–activity relationship between the catechol group in the B ring of these flavonoids with the enzyme was found [18]. Anti-inflammatory and antioxidant activity has also been observed for phenylpropanoid glycosides such as leucoseptoside, martynoside, and verbascoside, isolated from *Sideritis perfoliata* [19]. Diterpenes isolated from the essential oils of *Sideritis* spp. have shown antimicrobial activity in vitro [20]. Furthermore, antifeedant activity was observed by *ent*-kaurene diterpenes, such as sideroxol, linearol, and 7-epicandicandiol, isolated from the acetone extracts of different *Sideritis* species [21].

*Sideritis sipylea* Boiss. is an endemic plant of the Balkans that is distributed across specific islands of the North Aegean Region, i.e., Lesvos, Chios, Samos, and Ikaria, as well as in western Turkey. The plant is perennial, 20–60 cm high, branched, and densely addressed white or greyish tomentose, glandular. The flowering time is between June and September, with the species inhabiting mainly limestone slopes at altitudes between 200–1600 m. It is considered an endangered species in the North Aegean islands of Greece [22], because it is collected in large amounts from populations growing in the wild.

Its essential oil (EO) has a characteristic and strong scent, most probably attributed to the monoterpenes contained [9]. As revealed by previous studies on *S. sipylea* from Greece (i.e., Lesvos, Samos), the chemical composition of EO may vary according to the collection area [9,23]. The EO of *S. sipylea* from Lesvos has demonstrated in vitro antimicrobial activity against six bacteria and three pathogenic fungi, with stronger effects than those elicited by the EOs of *S. clandestina* subsp. *clandestina* and *S. raeseri* subsp. *attica* [9]. The in vitro antimicrobial potential of isolated compounds, mainly from the non-polar extracts (e.g., petroleum ether) of *S. sipylea* aerial parts collected in Turkey, has also been reported [24,25]. The most prominent effects were observed for kaurene diterpenes, such as 7-epicandicandiol. Regarding the polar extracts of *S. sipylea*, the methanolic extracts have shown comparatively strong antioxidant activity and high total phenolic content [26]. However, most studies so far have partially focused either on volatile/non-polar or polar extracts and constituents thereof, without providing further insight into the total chemical profile of this species and correlating it with different biological activities.

In continuation of our investigation on the ethnopharmacological properties of medicinal plants in the Greek islands of the North Aegean Region, we report herein the chemical profile of *S. sipylea*, considering both polar and non-polar constituents. Different extraction methods, specifically hydrodistillation (HD), supercritical fluid chromatography (SFE) and ultrasound-assisted extraction (UAE), were used, covering a wide range of polarities allowing at the same time a comparative study. For the analysis of the different extracts, gas chromatography-mass spectrometry (GC-MS) and ultra-performance liquid chromatography coupled with high-resolution mass spectrometry (UPLC-HRMS) were utilized accordingly. Finally, we examined whether the extracts derived from *S. sipylea* exhibit anti-tyrosinase and anti-elastase activity for the purpose of identifying anti-aging and skin-whitening properties, in addition to its well-known antioxidant capacity, investigating their potential to be used for dermo-cosmetic purposes.

## 2. Results and Discussion

### 2.1. Hydrodistillation and Extraction

In order to obtain the volatile and other non-polar compounds, but also the polar constituents of *S. sipylea*, three different techniques were utilized, in parallel. Specifically, hydrodistillation (HD) was employed for the recovery of the essential oil (EO), followed by supercritical fluid extraction (SFE), using supercritical CO_2_ (sCO_2_), and ultrasound-assisted extraction (UAE) with four different solvents. The extracts obtained by applying the aforementioned techniques are summarized in Table 1, accompanied by the respective extraction yields.

As shown in Table 1, the use of UAE, specifically the water/methanol (HA) and methanol (ME) extracts, presented the highest yields, compared to the extracts of lower polarities and/or obtained by other techniques. Moreover, the extraction yield of SFE surpassed the respective yields for the dichloromethane (DCM) extract and essential oil (EO). SFE can be regarded as a green and convenient alternative to the traditional techniques to recover non-polar compounds from aromatic plants, such as *Sideritis* spp. [27]. Moreover, due to the absence of organic solvents, it is compatible and preferable for pharma, food and cosmetic applications. However, it has not been utilized so far in place of hydrodistillation or extraction with non-polar solvents.

### 2.2. Chemical Analysis of Different Extracts

#### 2.2.1. GC-MS Analysis

**Figure 1:**
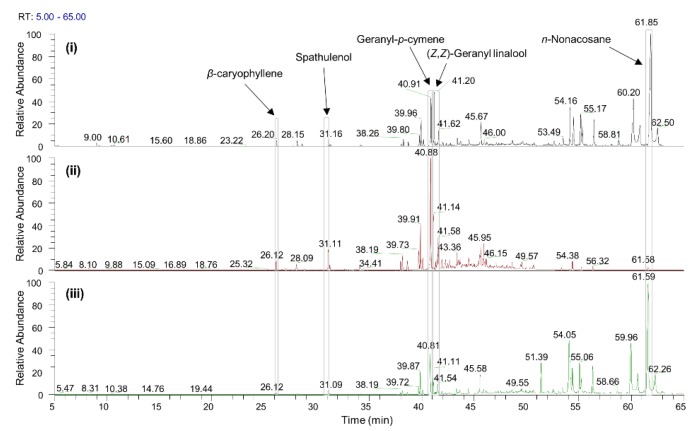
Gas chromatography-mass spectrometry (GC-MS) chromatograms of (**i**) dichloromethane (DCM) extract, (**ii**) essential oil (EO), and (**iii**) supercritical fluid extract (SFE) obtained from *S. sipylea*.

The chemical composition of the DCM extract, EO, and the SFE extract derived from *S. sipylea* was determined by GC-MS analysis (Figure 1). Listed in Table 2, in order of elution, are the compounds identified in the non-polar extracts examined.

Overall, 79 compounds were tentatively identified (Table 2) in the investigated DCM, EO, and SFE samples, representing the 79.43%, 83.32%, and 81.49% of the total peak area, respectively. Hydrocarbons were the most abundant compounds detected in all three non-polar extracts, with SFE presenting the highest amount (53.89%). This type of enrichment in hydrocarbon compounds, such as long-chain alkanes, may be explained, however, by the fact that the highest extraction yield (1.65% *w*/*w*) was observed in the case of SFE. Regarding the EO sample, sesquiterpene hydrocarbons, oxygenated sesquiterpenes, diterpene hydrocarbons, and oxygenated diterpenes were the major constituents (4.29%, 8.96%, 38.15%, and 29.80%, respectively). Monoterpene hydrocarbons such as α-pinene, β-pinene, sabinene, verbenol, and borneol were identified only in the DCM extract, representing a minor percentage (1.42%), unlike previous reports [9,23]. The bicyclic sesquiterpenes β-caryophyllene and caryophyllene oxide were present in all three different types of extracts (i.e., DCM, SFE, and EO), as shown in Figure 1, and have been investigated for potential activity as analgesic and anticancer agents [28]. These two bicyclic sesquiterpenes act as selective agonists of cannabinoid receptors (CB) and may exert a further use of *Sideritis* essential oil as an analgesic agent; however, the mechanism of action needs to be clarified. The presence of spathulenol (Figure S3) might be implicated in the reported antibacterial use of the essential oil from *S. sipylea* [25,29]. Moreover, geranyl linalool that is present in DCM, SFE, and EO is a common fragrance ingredient used in dermo-cosmetics [30]. Oxygenated diterpenes were the most abundant group of terpenoids detected in the *S. sipylea* non-polar extracts, especially in DCM and SFE extracts. It is well known that Mediterranean *Sideritis* spp. contain tetracyclic diterpenes of the *ent*-kaurane type [4]. Siderol, sideridiol, and 7-epicandicandiol were observed in all non-polar extracts of *S. sipylea*. The biological importance of these kaurane diterpenes isolated from this endemic species, with the use of eco-friendly technologies such as SFE, is among the aspects that merit further research. The aforementioned extract is mainly characterized by long-chain hydrocarbons (44.04%) and oxygenated diterpenes (26.23%). Those data for *S. sipylea* are reported here for the first time. It is worth noting that, in all previous research studies, we observed the phytochemical analysis of the essential oil of this endemic plant or the isolation of specific plant metabolites by employing solvent extraction and conventional chromatographic techniques [8,9,23,24]. Furthermore, the use of the promising SFE extraction technology has been reported so far only for *S. scardica* [27].

#### 2.2.2. UPLC-HRMS Analysis

**Figure 2:**
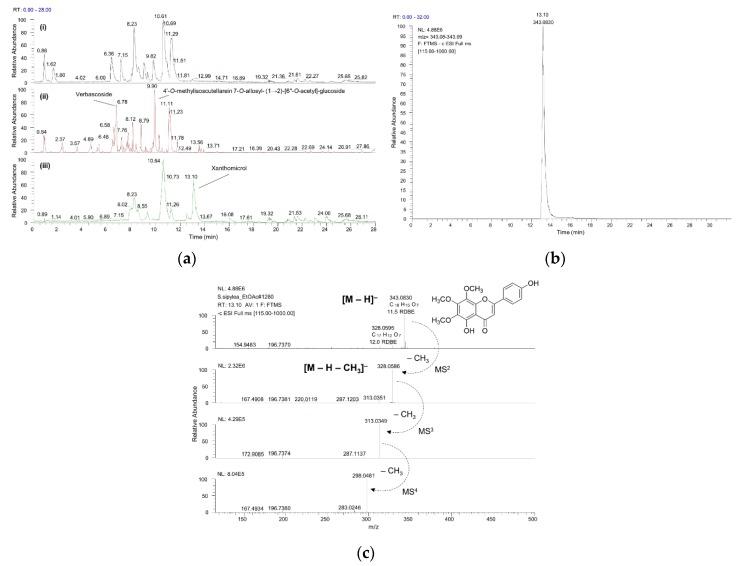
(**a**) UPLC-ESI(–)-HRMS chromatograms of (i) water/methanol (HA), (ii) methanol (ME), and (iii) ethyl acetate (EtOAc) total extracts of *S. sipylea*. (**b**) Extracted ion chromatogram (XIC) of xanthomicrol, detected mainly in the EtOAc extract. (**c**) High-resolution mass spectrometry (HRMS) spectra indicating the fragmentation pattern of xanthomicrol; full scan (top trace) and HRMS^2^ to HRMS^4^ spectra.

The chemical composition of the polar HA and ME extracts, together with the medium polar EtOAc extract of *S. sipylea*, was identified on the basis of UPLC-ESI-HRMS^n^ analysis, classifying the compounds into several chemical classes. The UPLC-HRMS chromatograms obtained by the analysis of the three extracts, using ESI(–) mode, are illustrated in Figure 2. The major compounds detected were hydroxycinnamic acid derivatives, phenylethanoid glycosides, and flavonoids, including flavonoid-7-*O*-diglycosides and flavonoid acetylglycosides. The identified components of the three extracts are listed in Table 2.

Chromatographic and spectrometric features such as retention time, suggested molecular formula, Ring Double Bond equivalents (RDBeq), as well as fragmentation pattern were used to identify individual compounds. A total of 33 components were detected and tentatively identified. As is depicted in Table 3, a large number of phenolic compounds was detected, especially in the HA and ME extracts. Specifically, seven different phenylethanoid glycosides (Supplementary Materials Figure S1) [4,5] were identified, such as echinacoside, forsythoside B, verbascoside, samioside, isoverbascoside, allysonoside, and leucoseptoside A, with verbascoside having the highest intensity (Figure 2a). Moreover, 17 different flavonoids were detected, with the flavonoid acetylglycoside 4′-*O*-methylisoscutellarein 7-*O*-allosyl- (1→2)-[6′′-*O*-acetyl]-glucoside having the highest intensity. Two iridoid glycosides, i.e., melittoside and its derivative, were detected mainly in the HA and ME extracts [31,32,33]. The presence of phenolic acids is limited to 6-*O*-caffeoyl-glucose, chlorogenic acid, and feruloylquinic acid. Chlorogenic acid has been previously identified as a component mainly present in *S. syriaca* from Crete as well as in *S. scardica* [32,34]. Nevertheless, its abundancy in many other plant species render it a weak chemical marker for specific *Sideritis* species.

In the EtOAc extract xanthomicrol [35], a trimethoxyflavone is one of the major secondary metabolites based on UPLC-HRMS analysis (Figure 2). It is well known that methoxylated flavones have promising pharmacological activities, such as antispasmodic, anti-platelet, and anti-cancer effects [36,37]. However, further investigations are needed in order to confirm that *S. sipylea* may exert the aforementioned effects.

### 2.3. Antioxidant Capacity

**Figure 3:**
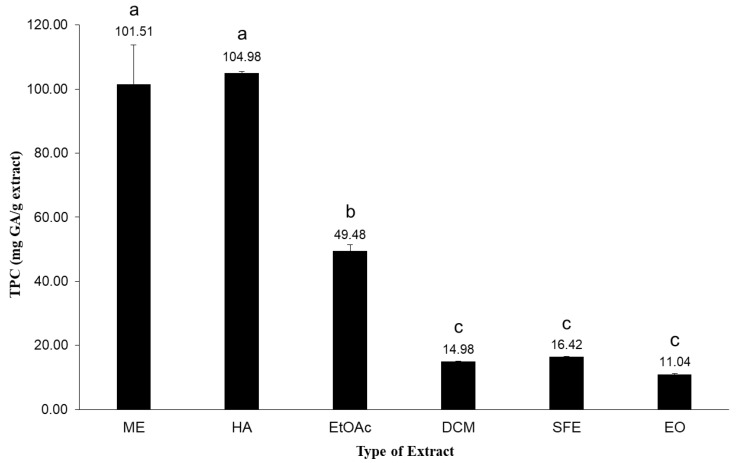
Total Phenolic Content (TPC) of the crude methanol (ME), water/methanol (HA), ethyl acetate (EtOAc), dichloromethane (DCM) and supercritical fluid (SFE) extracts, along with the essential oil (EO) obtained from *S. sipylea*. The values are the mean of three replicate determinations ± standard deviation. Different letters indicate significant differences among TPC values (*p* < 0.05).

Cellular oxidation is a complex biochemical condition induced by Reactive Oxygen Intermediates (ROI) and the byproducts generated from oxygen metabolism. The reactive species are derived from normal physiological and metabolic processes that are essential for the cell. In the plant kingdom, there are different compound classes that can act as antioxidants [38]. Flavonoids, such as flavonols, flavones, flavanones, isoflavonoids, and anthocyanidins, among others, have the ability to effectively moderate the harmful effects of Reactive Oxygen Species (ROS). Phenolic compounds constitute a well-known class of antioxidants in order to scavenge free radicals. The Total Phenolic Content (TPC) along with in vitro antioxidant activity assays, such as the 2,2-diphenyl-1-picrylhydrazyl (DPPH) free radical scavenging of the endemic *S. sipylea* in Turkey, has been previously reported, and, based on this study, the ME extract presented the highest TPC value, exceeding the respective value for the water extract [26]. It should be noted that TPC of hydroalcoholic extracts from *S. sipylea* was not reported therein. A higher ratio of phenolic compounds is extracted in more polar solvents, and this can explain the higher TPC values. Moreover, it is known that the TPC in plants depends on various factors, such as extraction solvents, handling and plant genetics [39]. So, we further investigated the TPC values of the ME, HA, EtOAc, DCM, and SFE extracts along with the EO of *S. sipylea*, by using the Folin–Ciocalteu method. TPC values were obtained from the calibration curve y = 0.0687x − 0.1348 with R^2^ = 0.9974, where x is the absorbance and y is the concentration of gallic acid solution (μg/mL) expressed as mg GAE/g of dry extract. The highest value of TPC was exhibited by the HA extract (104.98 ± 0.05 mg GAE/g), followed by the ME extract (101.51 ± 12.20 mg GAE/g), but with no significant difference between them (*p* = 0.9536). All other values are presented in Figure 3.

Higher values of TPC are associated with a higher content of phenolic compounds in the plant extracts. The phenolic compounds have a fundamental role in the plant biology and defense mechanisms [40]. It is well-known that the organoleptic properties of the plant, such as taste, smell and flavor are being modified by phenolic compounds [41]. Moreover, as mentioned above, they protect the plant from oxidative stress, microorganisms, and insects. A wide range of phenolic compounds was observed in the extracts. The EO obtained by HD and the non-polar fractions obtained by SFE and DCM contained much smaller amounts of phenolic compounds than the EtOAc, ME, and HA extracts. The comparative results concerning the whole spectrum of different extract polarities in *S. sipylea* have not been reported previously.

Moreover, the scavenging activity of these extracts was determined based on their DPPH neutralization. The DPPH radical scavenging is a widely used method to evaluate the ability of plant extracts to scavenge free radicals, generated from DPPH reagent. The effective concentration to reduce the DPPH radical to 50% (IC_50_) (defined as the concentration of substrate that causes a 50% loss of DPPH activity), was determined by plotting a linear regression curve of DPPH activity versus the ratio of sample concentration to DPPH, as previously reported [42]. The DPPH free radical scavenging effect of *S. sipylea* extracts was in the following order: SFE < DCM < EO. The lowest value of IC_50_ means a higher antioxidant potential of the plant extract. The most polar extracts (i.e., methanolic and hydroalcoholic) showed the lowest IC_50_ values (0.115 for ME and 0.116 for HA), while the highest IC_50_ value (0.202) and therefore the lowest antioxidant activity was presented for the essential oil (Supplementary Materials Figure S2).

Antioxidants with DPPH radical scavenging activity can donate hydrogen to free radicals, resulting in the inhibition of propagating the phase of lipid peroxidation. It is well established that free radical scavenging activity of plant extracts is mainly due to phenolic compounds [43]. The results clearly indicate that HA and ME extracts, which contained the highest amount of total phenolics, were the stronger radical scavengers.

### 2.4. Tyrosinase and Elastase Inhibitory Activity

**Figure 4:**
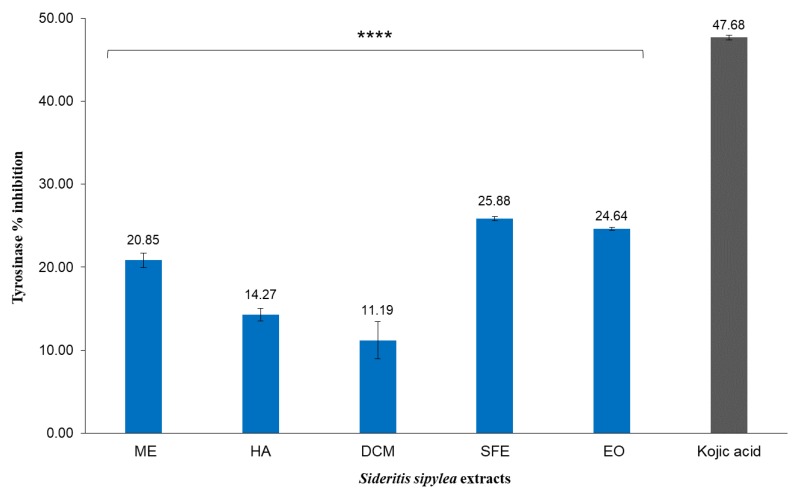
Tyrosinase inhibitory effect of the methanol (ME), water/methanol (HA), dichloromethane (DCM) and supercritical fluid (SFE) extracts, along with the essential oil (EO) of *S. sipylea* at 150 μg/mL. The values are the mean of three replicate determinations ± standard error. Kojic acid was used as a positive control at 150 μg/mL. Statistical analysis was performed by one-way ANOVA, and Dunnett’s test was employed to compare extracts with the positive control, with *p* < 0.0001 (****).

The inhibitory effect on tyrosinase of the two most polar ME and HA extracts, the non-polar DCM and SFE extracts, as well as the EO of *S. sipylea* was investigated at five different concentrations. Tyrosinase is a key regulatory enzyme that catalyzes melanin synthesis within melanocytes. Therefore, tyrosinase inhibitors have become increasingly important in cosmetics and pharmaceuticals as whitening agents [44]. Thus far, however, there have not been reports to confer the evaluation of the tyrosinase inhibitory activity of different polarity extracts from the aerial parts of *S. sipylea*. Figure 4 shows the results for tyrosinase inhibitory activity.

The tyrosinase inhibitory activity of the different extracts was enhanced with increasing concentration. The extracts examined show a maximum inhibition of ~26% at the concentration of 150 μg/mL, while the minimum percentage of inhibition is presented by the DCM extract with a value of ~11% (Figure 4). Previously, it was reported that the acetone and methanol extracts of *S. stricta* exhibited a 15.66% ± 0.11% and 23.29% ± 0.56% inhibition of tyrosinase, respectively, at 200 μg/mL [45]. By examining a lower concentration (150 μg/mL), all UAE extracts, SFE, and EO exhibited a promising inhibition of tyrosinase. Previously, several compounds isolated from *S. perfoliata,* including acteoside (verbascoside), ajugoside, caffeic acid, leucoseptoside A, and martynoside, showed tyrosinase and/or melanin production inhibition activity [46,47,48].

**Figure 5:**
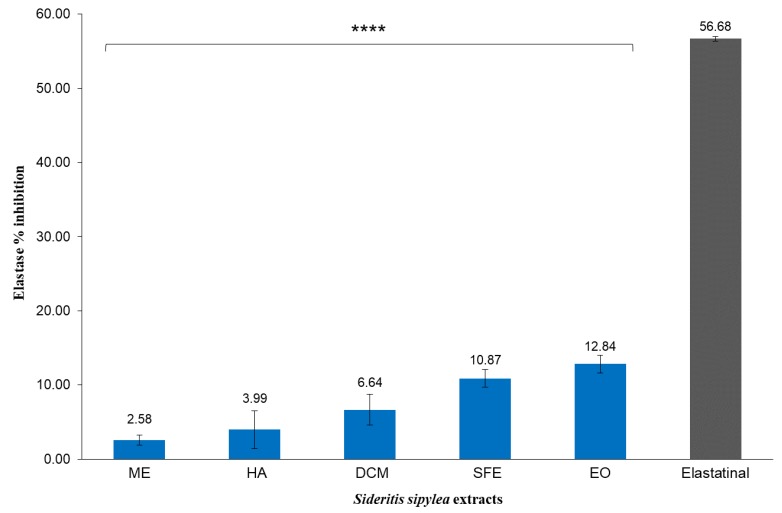
Elastase inhibitory effect of the methanol (ME), water/methanol (HA), dichloromethane (DCM), and supercritical fluid (SFE) extracts, along with the essential oil (EO) of *S sipylea* at 0.5 μg/mL. Values are mean of three replicate determinations ± standard error. Elastatinal was used as a positive control at 0.5 μg/mL. Statistical analysis was performed by one-way ANOVA, and Dunnett’s test was employed to compare extracts with the positive control, with *p* < 0.0001 (****).

The respective results for the elastase inhibitory activity of *S. sipylea* extracts are shown in Figure 5. Elastin is a highly elastic protein found in connective tissues and has a fundamental role in the tissue configuration. Side effects such as exposure to UV irradiation and oxidative damage upregulate the expression of elastase, a serine protease, which hydrolyzes the dermal elastin. This reduces skin elasticity and this effect may be associated with skin-aging. Inhibitors of elastase may have an anti-aging effect and their topical application to the surface of human skin may have beneficial effects, especially in the case of increased dryness [49].

By investigating the anti-elastase activity of *S. sipylea* extracts, all three non-polar extracts (DCM, SFE, and EO) showed an inhibitory effect on elastase (6.64, 10.87, and 12.84%, respectively) at 0.5 μg/mL, while the polar extracts ME and HA showed an inconsiderable effect (Figure 5). As regards the EO, at the concentration of 0.5 μg/mL, it showed the highest inhibitory effect on elastase (12.84%). This is a result that adds a value to the EO of *S. sipylea* to help prevent skin ageing, by using it in healing oils. The moderate enzyme inhibition may have the potential to maintain skin elasticity. According to the GC-MS results, the EO of *S. sipylea* is characterized by the presence of oxygenated compounds, including oxygenated sesquiterpenes and diterpenes. Such compounds have shown a moderate inhibition activity on the elastase enzyme explained by their direct binding with the enzyme [50]. However, in the present work we observed the inhibitory effect of the total EO, without considering the possible antagonism for the binding site, neither the synergistic effects of the compounds that may be responsible for the enzyme inhibition. Even if elastase inhibitory activity has been previously reported for the EtOH extract of *S. perfoliata* [51], no elastase inhibition has been reported before for *S. sipylea*.

The results also suggest that tyrosinase inhibition positively correlates with TPC and DPPH free radical scavenging activity, suggesting that flavonoid compounds present in *S. sipylea* are responsible for the tyrosinase inhibitory activity of the species. Furthermore, a correlation between the reduction in extract polarity and % elastase inhibition was observed at 500 μg/mL. The EO at the same concentration presented the highest value (%) of elastase inhibition (20.28%). A reduction in elastase activity up to 70% has been observed for the essential oils having eugenol, cinnamaldehyde, and geraniol [52]. From the data obtained in this study, and in comparison, with those reported in the literature, one can argue that the activities observed for the EO of *S. sipylea* are not only due to its major components but may also be due to synergistic effects with other minor components. Therefore, it can be argued that the EO of *S. sipylea* is a potential inhibitor of proteolytic enzymes and can be used in dermo-cosmetic formulations. However, further studies are needed for its stability in aqueous solutions and its toxicity.

*Sideritis* is a very common plant genus used in folk medicine and lately its extracts have become prevalent in many cosmetic formulations. Anti-aging and skin-whitening ingredients can be identified investigating different extracts of the endemic *Sideritis* species for their tyrosinase and elastase inhibitory activity. Both enzyme tests can be very useful in the search for active natural cosmeceuticals. The experimental results confirm many of the ethnopharmacological uses of this endemic *Sideritis* species, especially related to its biological properties, considering that, in the islands of the North Aegean Region wherein this species is grown (i.e., Lesvos, Chios, Ikaria, and Samos, Greece), it is commonly used as herbal tea. This study represents the first report on phytochemical composition, based on both GC-MS and UPLC-HRMS analyses, and the anti-aging and skin-whitening properties of *S. sipylea*. Further experiments should be conducted, however, in order to isolate and identify specific bioactive compounds from this species, as well as to stabilize the efficacy of non-toxic doses of the extracts and isolated compounds. Moreover, future studies could possibly employ more samples collected throughout the North Aegean Region, in order to explore the correlation between different chemotypes, habitats, climatic and pedological conditions [53,54].

## 3. Materials and Methods

### 3.1. Reagents and Materials

Methanol (MeOH), ethanol (EtOH, 96%), ethyl acetate (EtOAc), and dichloromethane (DCM) were purchased from Carlo Erba Reactifs SDS (Val de Reuil, France). Acetonitrile and formic acid of LC-MS grade were acquired from Fisher Scientific (Leicestershire, UK), while ultrapure water obtained from a Milli-Q^®^ purification system (Merck Millipore, Darmstadt, Germany) was used for LC-MS analysis as well as to prepare all aqueous solutions. 1,1-Diphenyl-2-picrylhydrazyl (DPPH), gallic acid, Folin & Ciocalteu’s phenol reagent 2M, and anhydrous sodium carbonate were purchased from Sigma-Aldrich (Saint-Quentin, France). Dimethyl sulfoxide (DMSO), mushroom tyrosinase (lyophilized powder, ≥1000 units/mg solid, EC Number: 1.14.18.1), 3,4-dihydroxy-L-phenylalanine, sodium phosphate monobasic, sodium phosphate dibasic, kojic acid, elastase type IV from porcine pancreas (EC Number: 3.4.21.36), *n*-Succinyl-Ala-Ala-Ala-*p*-nitroanilide (EC Number: 257-823-5), Trizma base (reagent grade), and elastatinal (microbial) were also purchased from Sigma-Aldrich (Saint-Quentin, France).

### 3.2. Plant Material

The aerial parts of *Sideritis sipylea* Boiss. were collected from the north Aegean island of Greece, Lesvos, Olympus mt., ca. 800 m. A voucher specimen (AXL035) is kept in the herbarium of the Faculty of Pharmacy, Department of Pharmacognosy and Natural Products Chemistry, National and Kapodistrian University of Athens, Greece. The air-dried material was powdered in a mill and portions thereof were subjected to hydrodistillation (HD), supercritical fluid extraction (SFE), and ultrasound-assisted extraction (UAE).

### 3.3. Hydrodistilation (HD) Procedure

The aerial parts (30 g) of *S. sipylea* were subjected to hydrodistillation for 3 h using a Clevenger-type apparatus. The distillation procedure enabled a 2-L round flask containing 1 L of distilled water, placed within a heating mantle. The resulting essential oil (EO) was dried over anhydrous sodium sulfate and stored at 4 °C until use. HD was performed in duplicate and the essential oil yield was estimated as 0.08% (*w*/*w*) of dried plant material.

### 3.4. Supercritical Fluid Extraction (SFE)

Extraction with supercritical carbon dioxide (SFE-CO_2_) was performed using laboratory-scale supercritical fluid equipment, model SFE 100 mL (SEPAREX, Champigneulles, France). The experiment was performed for 2 h by filling the stainless-steel extraction vessel (100 mL) with ca. 14 g of dried plant material. Extraction was carried out in duplicate using 100% CO_2_. The pressure in the extraction vessel was kept constantly at 300 bar, while the flow rate of CO_2_ was 25 g/min. The extraction and separation temperatures were set at 40 and 35 °C, respectively. Subsequently, the valve was opened every 20 min to partially collect the extract. Finally, the extract obtained was stored at 4 °C until use. The extraction yield was estimated as 1.65% (*w*/*w*) of the dried plant material.

### 3.5. Ultrasound-assisted Extraction (UAE)

The dried aerial parts of *S. sipylea* (10 g for each solvent) were extracted separately with dichloromethane (DCM), ethyl acetate (EtOAc), methanol (ME), and a mixture of distilled water/methanol (50:50 *v*/*v*) (HA). UAE was carried out within a PEX 3 Sonifier (REUS, Contes, France) of 3 L, composed of a stainless-steel reactor equipped with a double-layered mantle and a transducer operating at a frequency of 25 kHz, with a maximum input power of 150 W. The double-layered mantle allowed us to control the temperature of the medium by cooling with tap water. All extractions were performed in duplicate at ambient temperature, using 400 mL of solvent each time. After filtration, the collected extracts were concentrated to dryness and the residues were weighed, resulting in 0.112 g of DCM extract (1.12% *w*/*w*), 0.100 g of EtOAc extract (1.00% *w*/*w*), and 1.29 g of ME extract (12.90% *w*/*w*). The HA extract (1.46 g; 14.64 % *w*/*w*) was lyophilized using freeze-drying. All UAE extracts were stored at 4 °C until analysis and further use.

### 3.6. Analysis of S. sipylea Extracts

#### 3.6.1. Gas Chromatography-Mass Spectrometry (GC-MS)

The chemical composition of *S. sipylea* non-polar extracts, i.e., DCM, SFE, and EO, was determined by GC-MS. All analyses were carried out on a Finnigan Trace GC Ultra 2000 apparatus (Thermo Electron Corporation, USA), equipped with an AI 3000 autosampler. The GC system was coupled with a Finnigan Trace DSQ mass selective detector, operating with electron ionization in positive mode (70 eV) using the full scan mass range of *m*/*z* 40–400, while a Trace TR-5MS (Thermo Scientific, USA) capillary column (30 m × 0.25 mm i.d.; 0.25 μm film thickness) was used. The injector and detector temperatures were set at 220 and 260 °C, respectively. Helium was used as a carrier gas, at a flow rate of 1.0 mL/min. Diluted samples of 1 μL (2 mg/mL in DCM) were injected in splitless mode. The oven temperature was programmed to increase from 40 to 260 °C at a rate of 4 °C/min, and then held isothermally for 10 min; the total run time was 65 min. Xcalibur 2.0.7 software (Thermo Scientific) was used for the operation of the system as well as for data handling and processing. The retention indices (RI) of compounds were calculated according to the retention time of (C_8_–C_29_) *n*-alkanes. Identification was based on the comparison of their RI with those previously reported and by matching their mass spectra with those of Wiley 2007 and NIST 2011 libraries or literature data [55,56].

#### 3.6.2. Ultra-Performance Liquid Chromatography Coupled with High Resolution Mass Spectrometry (UPLC-HRMS)

UPLC-HRMS was employed with a view to investigate the chemical composition of *S. sipylea* extracts obtained with solvents of high or medium polarity, i.e., HA, ME, and EtOAc extracts. The analyses were performed using an Acquity H-Class UPLC system (Waters Corp., Milford, USA), equipped with a quaternary pump, an autosampler, an online vacuum degasser, and a temperature-controlled column and sample compartment, and hyphenated to a hybrid LTQ-Orbitrap Discovery XL (Thermo Scientific, Brehmen, Germany) mass spectrometer with an electrospray ionization (ESI) source. Xcalibur 2.0.7 (Thermo Scientific) software was used for data acquisition and processing. For all analyses, the samples were diluted at a concentration of 200 μg/mL, using MeOH/H2O 60:40 (*v*/*v*) for both type of extracts. A Fortis C_18_ (Fortis Technologies Ltd., Cheshire, UK) column (100 × 2.1 mm, 1.7 μm) was used with a mobile phase consisted of water (solvent A) and acetonitrile (solvent B), both containing 0.1% formic acid (*v*/*v*). The gradient program was as follows: initially 5% (B) maintained for 3 min, then increased until reaching 100% (B) in 18 min, maintained at 100% (B) for 2 min, and finally decreased to 5% (B) in 2 min and held at the initial conditions (7 min) for re-equilibration, with a total run time of 32 min. The flow rate was 0.4 mL/min, while the injection volume was 10 μL. Column temperature was set at 40 °C.

MS data acquisition was performed in negative (ESI–) ionization mode, in the full scan mass range of *m*/*z* 115.0–1000.0, using a resolution of 30,000. The capillary temperature was set at 350 °C. The tuning of capillary voltage and tube lens was at −30 and −100 V, respectively. The source voltage was set at 2.70 kV (ESI–), while source current was 100 μA.

The MS^2^ spectra were recorded using data-dependent acquisition, with a collision-induced dissociation (CID) value of 35% and a mass resolution of 7,500. Nitrogen with a flow rate set at 40 and 10 arbitrary units was used as sheath gas and auxiliary gas, respectively.

### 3.7. Assessment of the Total Phenolic Content (TPC) of Extracts

The TPC values of the different *S. sipylea* extracts was determined using the Folin–Ciocalteu reagent, as previously described [57]. A total of 20 μL of extract was added to a tube containing 1 mL distilled water, followed by the addition of 100 μL of Folin-Ciocalteu reagent and incubation for 3 min at room temperature. Subsequently, 280 μL of 25% *w*/*v* sodium carbonate (Na_2_CO_3_) solution, along with 600 μL of distilled water, was added to the mixture. Finally, following 1 h incubation at room temperature in the dark, the absorbance was measured at 765 nm versus a blank lacking the extract. The measurement was carried out on a Hitachi U-1900 radio beam spectrophotometer (serial no. 2023–029; Hitachi Ltd., Tokyo, Japan). The optical density of the sample without the Folin–Ciocalteu reagent at 765 nm was also measured. TPC was determined using a gallic acid standard curve (50–1500 μg/mL) and is presented as µg of gallic acid equivalents per mg of extract.

### 3.8. DPPH^•^ Radical Scavenging Assay

The DPPH radical scavenging assay (RSC) was performed according to a previously described method [42]. Briefly, 1 mL of freshly prepared ethanolic solution of DPPH radical (100 μM) was mixed with the tested extract solution in EtOH at various concentrations (0.5, 1.0, 2.0, and 4.0 mg/mL). The mixture was then vortexed and incubated at room temperature in the dark for 30 min, followed by absorbance measurement at 517 nm on the reader Infinite^®^ 200 PRO series (Tecan Group Ltd., Männedorf, Switzerland). Experiments were performed in triplicate for each sample and twice in total. Gallic acid was used as a positive control at a concentration of 30 μM. A negative control with 10 μL DMSO and 190 μL DPPH was performed each time. The blank solution contained 190 μL EtOH and 10 μL sample. The radical scavenging activity percentage (AA%) was calculated as follows: AA% = [1 − ((A_sample_ − A_blank_)/A_control_)] × 100, where A_control_ is the absorbance of the negative control, A_sample_ is the absorbance after the reaction of samples with DPPH, and A_blank_ is the absorbance of sample with EtOH instead of DPPH. Moreover, the IC_50_ value indicating the extract amount that caused 50% scavenging of the DPPH radical was calculated.

### 3.9. Tyrosinase Inhibition Activity

All extracts of *S. sipylea* were evaluated at 150 μg/mL (final concentration in the well). The capacity of the samples to inhibit the catalytic action of tyrosinase in the oxidation of L-DOPA to dopachrome was determined by an enzymatic method, as described previously [58]. The activity of tyrosinase was measured at 475 nm using the Infinite^®^ 200 PRO plate reader. The inhibitory potency of the samples against this enzyme was compared with that of the positive control, kojic acid (IC_50_ = 495.5 μM), known as a strong tyrosinase inhibitor. In a 96-well microplate, 40 μL of the tested sample (dissolved in the PBS buffer from 1.5 to 0.3 mg/mL) and 40 μL of mushroom tyrosinase were mixed and incubated for 10 min at room temperature avoiding light exposure. Then, 40 μL of 2.5 mM L-DOPA dissolved in buffer was added and the measurement of dopachrome formation at 475 nm was done. The final DMSO concentrations did not exceed 5% of the total volume. The inhibition percentage was calculated as follows: Inhibition (%) = [((A_control_ − A_control’s blank_) − (A_sample_ − A_sample’s blank_)) / (A_control_ − A_control’s blank_)] × 100, where A_control_ is the absorbance of the mixture of buffer, tyrosinase, sample solvent, and substrate and A_sample_ is the absorbance of the mixture of buffer, tyrosinase, samples or kojic acid solution, and substrate.

### 3.10. Elastase Inhibition Activity

The porcine pancreatic elastase type IV (PPE), a lyophilized powder at ≥4 units/mg protein, was used for this bioassay. ME and HA crude extracts of *S. sipylea* were evaluated at concentrations of 500 μg/mL (final concentration in the well). PPE inhibition was tested according to a method described previously [59], using *n*-succinyl-Ala-Ala-Ala-*p*-nitroanilide as a substrate, and monitoring the release of *p*-nitroaniline. The amount of *p*-nitroaniline was determined spectrophotometrically at 405 nm. The reaction mixture contained 80 μL of Trizma-base buffer (50 mM, pH 7.5), 10 μL of sample (500 μg/mL in buffer) and 5 μL of elastase. The final solutions were incubated for 15 min at room temperature in the dark. Afterwards, 15 μL of 2 mM N-succinyl-Ala-Ala-Ala-*p*-nitroanilide dissolved in Trizma buffer was added and the solutions were incubated for 30 min at 37 °C. Elastatinal is used as a positive control (IC_50_ = 0.5 μg/mL). All experiments were performed in triplicate and absorbance was measured using the Infinite^®^ 200 PRO plate reader. The % inhibition of elastase was calculated by the formula Inhibition (%) = (A_control_ − A_sample_) / A_control_) × 100, where A_control_ is the absorbance of the solution containing the buffer, elastase, the sample solvent and the substrate at 405 nm, and A_sample_ is the absorbance of the buffer, elastase, sample or elastinal and substrate mixture at 405 nm. For each sample, a blank experiment was performed.

### 3.11. Statistical Analysis

The values are represented as mean ± SE (*n* ≥ 3) for all enzymatic bioassays, while, for DPPH and TPC assays, the results are reported as mean ± SD (*n* ≥ 3). By using one-way ANOVA, the occurrence of statistical differences among the data was evaluated. To compare samples with the positive control, multiple comparisons of means were performed using Dunnett’s test (*p* values of < 0.05 (*), < 0.01 (**), < 0.001 (***), and < 0.0001 (****), respectively); otherwise, means were separated using Tukey’s test (*p* < 0.05). GraphPad Prism 6.01 (GraphPad Software Inc., California, CA, USA) software was used for all the analyses.

## 4. Conclusions

The overall aim of this study was to thoroughly investigate the total phytochemical profile and the biological activity of different extracts of the endemic and endangered species *Sideritis sipylea* Boiss. Promising results were obtained with a view to find new active ingredients for cosmetic formulations in accordance to its well-known antioxidant and anti-inflammatory properties. These findings reinforce the ethnopharmacological use references for this species in Greece and Turkey, especially for the most polar extracts, such as the one obtained using water/methanol. Therefore, these fractions of *S. sipylea* may be a novel source for the development of new dermo-cosmetic agents.

## Figures and Tables

**Table 1:** *S. sipylea* extracts of different polarity and their % (*w*/*w*) yields.

Type of Extract	Solvent	Extraction Technique	Yield (%*w*/*w*) ^1,2^
Essential oil (EO)	–	HD	0.08 ± 0.02 ^d^
sCO_2_ extract (SFE)	sCO_2_	SFE	1.65 ± 0.09 ^c^
DCM extract (DCM)	DCM	UAE	1.12 ± 0.12 ^cd^
EtOAc extract (EtOAc)	EtOAc	UAE	1.00 ± 0.08 ^cd^
MeOH extract (ME)	Methanol	UAE	12.90 ± 0.60 ^b^
H_2_O/MeOH (50:50 *v*/*v*) (HA)	Water/Methanol	UAE	14.64 ± 0.52 ^a^

^1^ Mean ± standard deviation (*n* = 2). ^2^ Means with no superscript letter in common are significantly different (Tukey’s test; *p* < 0.05).

**Table 2:** GC-MS analysis of *S. sipylea* non-polar extracts obtained using ultrasound-assisted extraction with dichloromethane (DCM), supercritical fluid extraction (SFE), and hydrodistillation (EO).

No.	RI ^1^	Compound	% Peak Area ^2^		
			DCM	EO	SFE
1	965	α-Pinene	0.95 ± 0.13	-	-
2	988	Sabinene	0.10 ± 0.01	-	-
3	991	β-Pinene	0.37 ± 0.03	-	-
4	1082	*cis*-Sabinene hydrate	0.03 ± 0.00	-	-
5	1107	Linalool	-	0.03 ± 0.01	-
6	1123	*trans*-*p*-Mentha-2,8-dien-1-ol	0.05 ± 0.00	-	-
7	1141	α-Campholenal	0.02 ± 0.01	-	-
8	1154	*trans*-Pinocarveol	0.02 ±0.00	0.04 ± 0.00	-
9	1158	*cis*-Verbenol	0.04 ± 0.00	0.06 ± 0.01	-
10	1185	Borneol	0.02 ± 0.01	0.04 ± 0.02	-
11	1191	Terpinen-4-ol	-	0.07 ± 0.02	-
12	1208	Myrtenol	0.03 ± 0.00	0.11 ± 0.02	-
13	1289	Bornyl acetate	0.02 ± 0.00	0.03 ± 0.00	-
14	1333	δ-Elemene	0.03 ± 0.00	0.03 ± 0.00	-
15	1340	α-Terpinyl acetate	-	0.03 ± 0.01	-
16	1374	α-Copaene	0.01 ± 0.00	0.03 ± 0.01	-
17	1381	(*E*)-β-Damascenone	-	0.07 ± 0.03	-
18	1386	β-Elemene	0.03 ± 0.00	0.10 ± 0.02	-
19	1417	β-Caryophyllene	0.57 ± 0.02	1.32 ± 0.34	0.20 ± 0.06
20	1435	(*Z*)-β-Farnesene	0.04 ± 0.00	0.17 ± 0.03	-
21	1445	(*E*)-β-Farnesene	0.08 ± 0.00	0.54 ± 0.11	-
22	1453	α-Humulene	0.02 ± 0.00	0.08 ± 0.02	0.04 ± 0.01
23	1461	α-Acoradiene	0.03 ± 0.00	0.08 ± 0.01	-
24	1465	9-epi-(*E*)-Caryophyllene	0.04 ± 0.01	0.11 ± 0.02	0.02 ± 0.01
25	1475	*ar*-Curcumene	-	0.19 ± 0.04	-
26	1477	Germacrene D	0.58 ± 0.01	0.97 ± 0.06	0.16 ± 0.04
27	1487	α-Zingiberene	-	0.12 ± 0.01	-
28	1490	Bicyclogermacrene	0.21 ± 0.01	0.11 ± 0.01	0.07 ± 0.03
29	1499	β-Bisabolene	-	0.11 ± 0.03	-
30	1500	β-Curcumene	0.04 ± 0.01	0.22 ± 0.04	-
31	1511	δ-Cadinene	-	0.06 ± 0.03	-
32	1531	(*E*)-γ-Bisabolene	-	0.05 ± 0.02	-
33	1536	*cis*-Sesquisabinene hydrate	0.07 ± 0.00	0.08 ± 0.01	0.05 ± 0.02
34	1575	Spathulenol	0.66 ± 0.04	3.02 ± 0.42	0.41 ± 0.05
35	1579	Caryophyllene oxide	0.17 ± 0.01	0.73 ± 0.06	0.14 ± 0.06
36	1606	Humulene epoxide II	-	0.07 ± 0.01	0.02 ± 0.01
37	1630	Isospathulenol	0.04 ± 0.00	0.19 ± 0.05	-
38	1636	α-Acorenol	0.02 ± 0.00	0.07 ± 0.02	-
39	1641	β-Acorenol	0.03 ± 0.01	0.02 ± 0.00	-
40	1645	*allo*-Aromadendrene epoxide	-	0.16 ± 0.01	-
41	1667	epi-β-Bisabolol	-	0.17 ± 0.05	-
42	1680	α-Bisabolol	0.17 ± 0.00	0.94 ± 0.20	-
43	1691	Amorpha-4,9-dien-2-ol	0.03 ± 0.00	0.12 ± 0.03	-
44	1705	(2*E*,6*Z*)-Farnesal	-	0.18 ± 0.04	-
45	1726	(*E*)-Sesquilavandulyl acetate	0.03 ± 0.01	-	0.02 ± 0.01
46	1825	Cyclopentadecanolide	0.04 ± 0.01	0.06 ± 0.00	0.07 ± 0.00
47	1835	Hexahydrofarnesyl acetone	0.26 ± 0.01	1.16 ± 0.00	0.20 ± 0.04
48	1843	(*Z*)-Lanceol acetate	0.68 ± 0.02	1.99 ± 0.03	0.46 ± 0.08
49	1905	Isopimara-9(11),15-diene	0.97 ± 0.05	2.52 ± 0.22	0.70 ± 0.12
50	1914	Totarene	2.37 ± 0.18	6.22 ± 0.34	2.18 ± 0.22
51	1923	Beyerene	0.56 ± 0.03	1.66 ± 0.22	0.40 ± 0.04
52	1949	Geranyl-α-terpinene	0.46 ± 0.00	0.62 ± 0.08	0.11 ± 0.02
53	1954	Geranyl-*p*-cymene	4.46 ± 0.16	17.49 ± 1.37	4.66 ± 0.33
54	1967	(*Z*,*Ζ*)-Geranyl linalool	5.20 ± 0.23	8.06 ± 0.63	2.76 ± 0.40
55	1976	Dolabradiene	0.15 ± 0.02	0.75 ± 0.15	0.22 ± 0.01
56	1981	Sclarene	0.30 ± 0.01	1.30 ± 0.12	0.26 ± 0.06
57	1987	(*E*,*Ζ*)-Geranyl linalool	1.56 ± 0.11	4.87 ± 0.16	1.31 ± 0.10
58	2003	(*Ζ*,*E*)-Geranyl linalool	0.47 ± 0.04	1.43 ± 0.08	0.32 ± 0.05
59	2015	13-epi-Dolabradiene	0.28 ± 0.01	1.88 ± 0.10	0.21 ± 0.04
60	2028	13-epi-Manool oxide	-	0.66 ± 0.08	0.09 ± 0.01
61	2033	(*E*,*E*)-Geranyl linalool	0.34 ± 0.01	1.60 ± 0.15	0.28 ± 0.06
62	2059	Manool	0.72 ± 0.04	2.17 ± 0.25	0.63 ± 0.12
63	2071	13-epi-Manool	0.52 ± 0.05	1.67 ± 0.13	0.46 ± 0.08
64	2105	Phytol	0.58 ± 0.02	1.46 ± 0.22	0.53 ± 0.11
65	2158	Abienol	2.42 ± 0.13	1.68 ± 0.40	2.18 ± 0.38
66	2173	Abieta-8(14),13(15)-diene	0.72 ± 0.02	5.71 ± 0.88	0.62 ± 0.08
67	2185	Sandaracopimarinal	0.50 ± 0.01	1.73 ± 0.33	0.51 ± 0.02
68	2238	Sclareol	0.19 ± 0.02	0.24 ± 0.08	0.19 ± 0.06
69	2259	7-α-hydroxy-Manool	0.18 ± 0.03	0.54 ± 0.11	0.21 ± 0.01
70	2301	3-α-hydroxy-Manool	0.61 ± 0.04	0.76 ± 0.16	0.36 ± 0.05
71	2343	Isopimarol	0.29 ± 0.01	0.69 ± 0.18	0.21 ± 0.02
72	2580	Sideridiol	4.77 ± 0.66	0.22 ± 0.03	5.92 ± 0.88
73	2584	*n*-Hexacosane	-	-	8.87 ± 0.45
74	2596	7-Epicandicandiol	3.53 ± 0.05	1.48 ± 0.17	3.89 ± 0.28
75	2639	Siderol	2.43 ± 0.32	0.54 ± 0.14	1.88 ± 0.30
76	2694	*n*-Heptacosane	3.63 ± 0.28	0.63 ± 0.15	3.91 ± 0.38
77	2802	*n*-Octacosane	0.97 ± 0.06	0.06 ± 0.02	1.09 ± 0.16
78	2929	*n*-Nonacosane	29.38 ± 1.75	0.95 ± 0.08	30.17 ± 2.03
79	2958	Sidol	5.34 ± 0.52	-	4.50 ± 0.35
Hydrocarbon compounds			47.35	44.08	53.89
Oxygenated compounds			32.08	39.24	27.60
Monoterpene hydrocarbons			1.42	-	-
Oxygenated monoterpenes			0.23	0.41	-
Sesquiterpene hydrocarbons			1.68	4.29	0.49
Oxygenated sesquiterpenes			2.20	8.96	1.37
Diterpene hydrocarbons			10.27	38.15	9.36
Oxygenated diterpenes			29.65	29.80	26.23
Norisoprenoids			-	0.07	-
Alkanes			33.98	1.64	44.04
Total identified (%)			79.43	83.32	81.49

^1^ Retention indices (RI) determined in reference to *n*-alkanes (C_8_–C_29_) on a non-polar TR-5 ms column. ^2^ Data are presented as mean ± standard deviation (*n* = 2).

**Table 3:** Chemical components identified by UPLC-ESI(–)-HRMS in the methanol (ME), water/methanol (HA) and ethyl acetate (EtOAc) extracts of *S. sipylea*.

No.	Compound	*t*_R_ (min)	[M − H]^−^ *m*/*z*	Error	Suggested	HRMS^2^ [M − H]^−^	*S. sipylea*			Ref.
				(ppm)	Formula		**ME**	**HA**	**EtOAc**	
1	6-*O*-Caffeoyl-glucose	0.84	341.1095	1.57	C_12_H_21_O_11_	**179** *	+	+	-	-
2	Quinic acid	0.88	191.0568	3.71	C_7_ H_11_ O_6_	173, **127**, 85	+	+	-	-
3	Melittoside	2.37	523.1672	0.64	C_21_H_31_O_15_	478, 457, 287, **197**	+	+	-	[31]
4	Melittoside derivative	2.37	569.1726	0.51	C_22_H_33_O_17_	**523**, 179	+	+	+	-
5	Unknown	3.57	375.1297	0.16	C_16_H_23_O_10_	**213**, 169, 151	+	+	-	-
6	Chlorogenic acid	4.69	353.0880	0.50	C_16_H_17_O_9_	**191**, 179	+	+	-	[32]
7	Unknown	5.36	435.1512	0.81	C_18_H_27_O_12_	**389**, 287, 197	+	+	+	-
8	Feruloylquinic acid	6.03	367.1036	0.50	C_17_H_19_O_9_	287, **191**	+	+	-	[4]
9	Echinacoside	6.46	785.2509	−0.06	C_35_H_45_O_20_	**623**, 461	+	+	-	[4]
10	Forsythoside B	6.58	755.2408	0.58	C_34_H_43_O_19_	623, **593**, 461	+	+	-	[32]
11	Verbascoside	6.78	623.1992	1.63	C_29_H_35_O_15_	477, **461**, 315	+	+	+	[4]
12	Samioside	6.91	755.2408	0.58	C_34_H_43_O_19_	623, **593**, 461	+	+	-	[4]
13	Apigenin 7-*O*-allosyl(1→2)glucoside	6.97	593.1518	1.01	C_27_H_29_O_15_	431, **269**	+	+	-	[33]
14	Isoscutellarein 7-*O*-allosyl(1→2)glucoside	7.11	609.1471	1.61	C_27_H_29_O_16_	447, 429, **285**	+	+	-	[4]
15	Isoverbascoside	7.18	623.1989	1.13	C_29_H_35_O_15_	**461**, 315	+	+	+	[4]
16	Allysonoside	7.39	769.2563	0.31	C_35_H_45_O_19_	637, **593**, 575, 461	+	+	-	[4]
17	Hypolaetin 7-*O*-[6′’’-*O*-acetyl]-allosyl(1→2)glucoside	7.41	667.1517	0.12	C_29_H_31_O_18_	**625**, 463, 301	+	+	-	[32]
18	Leucoseptoside A	7.66	637.2142	0.69	C_30_H_37_O_15_	491, **461**, 443	+	+	-	[4]
19	Apigenin 7-*O*-glucoside	7.76	431.0990	1.53	C_21_H_19_O_10_	**269**	+	+	-	[4]
20	Apigenin 7-*O*-[6′’-*O*-acetyl]-allosyl(1→2)glucoside	8.00	635.1625	1.14	C_29_H_31_O_16_	593, 515, **269**	+	+	-	[33]
21	Luteolin 7-*O*-allosyl-(1→2)-[6′’-*O*-acetyl]-glucoside	8.12	651.1570	0.47	C_29_H_31_O_17_	591, 429, **285**	+	+	+	[32]
22	3′-*O*-Methylhypolaetin 7-*O*-[6′’’-*O*-acetyl]-allosyl(1→2)glucoside	8.41	681.1679	0.90	C_30_H_33_O_18_	**639**, 621, 459, 315	+	+	+	[4]
23	4′-*O*-Methylisoscutellarein 7-*O*-allosyl(1→2)glucoside	8.79	623.1625	1.26	C_28_H_31_O_16_	461, 443, **299**, 284	+	+	+	[4]
24	Martynoside	9.33	651.2297	0.46	C_31_H_39_O_15_	505, **475**, 457	+	+	+	[5,33]
25	4′-*O*-Methylisoscutellarein 7-*O*-allosyl-(1→2)-[6′’-*O*-acetyl]-glucoside	9.96	665.1728	0.70	C_30_H_33_O_17_	623, 461, **299**	+	+	+	[4,32]
26	Isoscutellarein 7-*O*-[6′’’-*O*-acetyl]-allosyl-(1→2)-[6′’-*O*-acetyl]-glucoside	9.98	693.1644	−4.14	C_31_H_33_O_18_	651, 633, 471, **285**	+	-	-	[32]
27	Apigenin 7-(6′’-*p*-coumaroylglucoside)	10.28	577.1358	1.02	C_30_H_25_O_12_	431, 413, 307, **269**	+	+	-	[32]
28	Apigenin 7-(4′’-*p*-coumaroylglucoside)	11.11	577.1356	0.82	C_30_H_25_O_12_	431, 413, 307, **269**	+	+	+	[4,32]
29	4′-*O*-Methylisoscutellarein 7-*O*-[6′’’-*O*-acetyl]-allosyl-(1→2)-[6′’-*O*-acetyl]-glucoside	11.78	707.1829	0.05	C_32_H_35_O_18_	665, 647, **299**, 284	+	+	+	[4]
30	Sideritoflavone	12.04	359.0776	0.86	C_18_H_15_O_8_	**344**	-	-	+	[5]
31	Luteolin derivative	12.54	313.0722	1.40	C_17_H_13_O_6_	**298**	+	-	+	-
32	Xanthomicrol	13.10	343.0826	0.74	C_18_H_15_O_7_	**328**, 313	-	-	+	[5,20]
33	Unknown	13.56	723.1722	0.35	C_39_H_31_O_14_	577, 559, **453**, 269	+	+	-	-

* Numbers in bold indicate the base peak ions according to the HRMS^2^ data.

## Supplementary Materials

The following are available online, Figure S1: Identified phenylethanoid glycosides in *S. sipylea* (feru-feruoyl, caff-caffeoyl, glc-glucose, api-apiose), Figure S2: IC_50_ values of crude methanol (ME), water/methanol (HA), ethyl acetate (EtOAc), dichloromethane (DCM), and supercritical fluid (SFE) extracts, along with essential oil (EO) obtained from *S. sipylea* aerial parts (mean ± SD). Significant differences among the IC_50_ values of extracts are indicated by different letters (*p* < 0.05), Figure S3: Fragment ions and relative abundance (*m*/*z*, %) of some characteristic volatile compounds identified in *S. sipylea* non-polar extracts. (a) β-Caryophyllene (% score of similarity: 35.05). tR: 26.1 min, EI-MS *m*/*z* (%): 41 (85), 53 (23), 55 (28), 67 (42), 69 (100), 77 (36), 79 (59), 81 (28), 91 (63), 93 (71), 105 (58), 106 (29), 107 (40), 119 (28), 120 (25), 133 (64), 147 (20), 161 (21), 204 (M^+^, 3). (b) Spathulenol (% score of similarity: 48.44). tR: 31.1 min, EI-MS *m*/*z* (%): 41 (64), 43 (100), 55 (22), 67 (33), 69 (44), 71 (29), 77 (30), 79 (38), 91 (55), 93 (41), 105 (58), 106 (25), 107 (38), 119 (48), 131 (26), 145 (21), 147 (24), 159 (34), 162 (22), 205 (38), 220 (M^+^, 2). (**c**) Caryophyllene oxide (% score of similarity: 35.01). tR: 31.2 min, EI-MS *m*/*z* (%): 41 (100), 43 (98), 53 (26), 55 (40), 67 (57), 69 (90), 71 (28), 77 (45), 79 (82), 81 (38), 91 (65), 93 (68), 95 (49), 96 (30), 105 (53), 106 (42), 107 (49), 109 (45), 110 (23), 121 (26), 220 (M^+^, 1). (d) Geranyl-*p*-cymene (% score of similarity: 92.68). tR: 40.9 min, EI-MS *m*/*z* (%): 41 (43), 67 (16), 69 (62), 91 (22), 95 (12), 105 (57), 109 (18), 117 (18), 119 (100), 120 (12), 123 (12), 131 (13), 132 (81), 133 (16), 145 (66), 159 (13), 227 ([M−CH_3_]^+^, 15).
