# Supplementary material for: Phytochemical Profile and Biological Activity of Endemic Sideritis sipylea Boiss. in North Aegean Greek Islands
Source: Molecules. 2020 Apr 26;25(9):2022. doi: 10.3390/molecules25092022 (PMC7248978; doi:10.3390/molecules25092022)
Supplement: Supplementary file 1 [file molecules-25-02022-s001.pdf]

Supplementary Material

Phytochemical Profile and Biological Activity of Endemic *Sideritis sipylea* Boiss. in North Aegean Greek Islands

Evangelos Axiotis, Eleftherios A. Petrakis, Maria Halabalaki and Sofia Mitakou *

Department of Pharmacognosy and Natural Products Chemistry, Faculty of Pharmacy, National and Kapodistrian University of Athens, Panepistimiopolis Zografou, 15771 Athens, Greece; axiotisevan@pharm.uoa.gr (E.A.); epetrakis@pharm.uoa.gr (E.A.P.); mariahal@pharm.uoa.gr (M.H.)
* Correspondence: mitakou@pharm.uoa.gr; Tel.: +30-210-727-4594

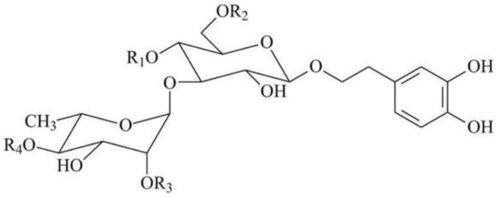

Compound	R_1	R_2	R_3	R_4
**echinacoside**	feru.	glc	H	H
**forsythoside B**	caff.	api.	H	H
**verbascoside**	caff.	H	H	H
**samioside**	caff.	H	H	api.
**isoverbascoside**	H	caff.	H	H
**allysonoside**	feru.	api.	H	H
**leucoseptoside A**	feru.	H	H	H

Figure S1. Identified phenylethanoid glycosides in *S. sipylea* (feru-feruoyl, caff-caffeoyl, glc-glucose, api-apiose).

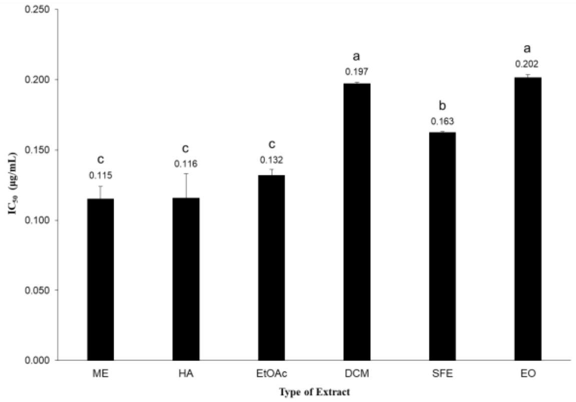

Figure S2. IC_{50} values of crude methanol (ME), water/methanol (HA), ethyl acetate (EtOAc), dichloromethane (DCM), and supercritical fluid (SFE) extracts, along with essential oil (EO) obtained from *S. sipylea* aerial parts (mean ± SD). Significant differences among the IC_{50} values of extracts are indicated by different letters ($p < 0.05$).

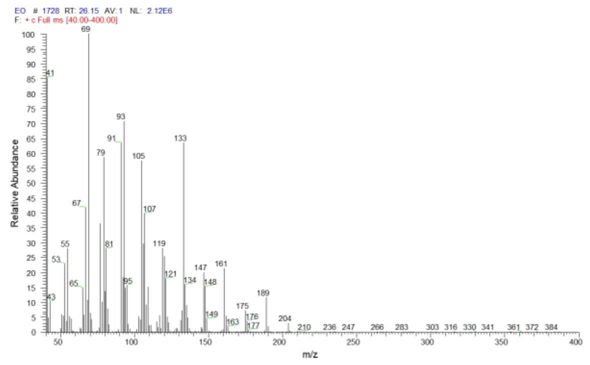

(**a**)

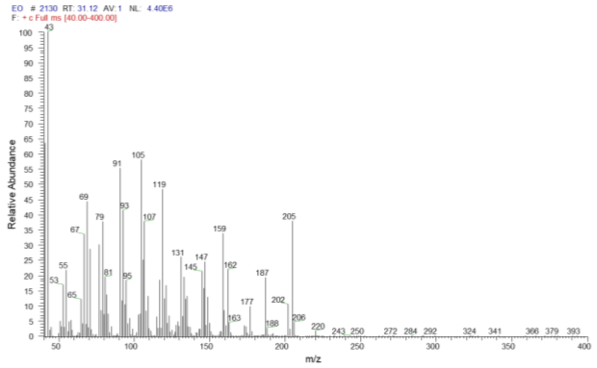

(**b**)

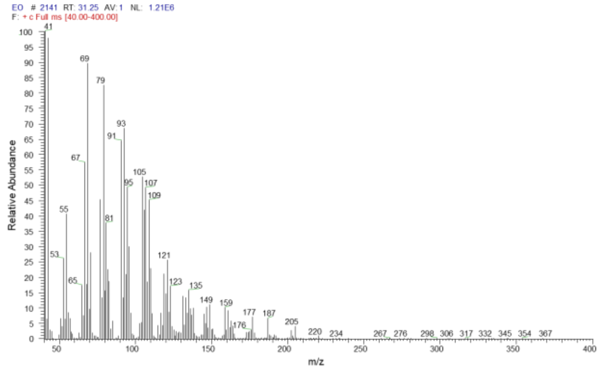

(**c**)

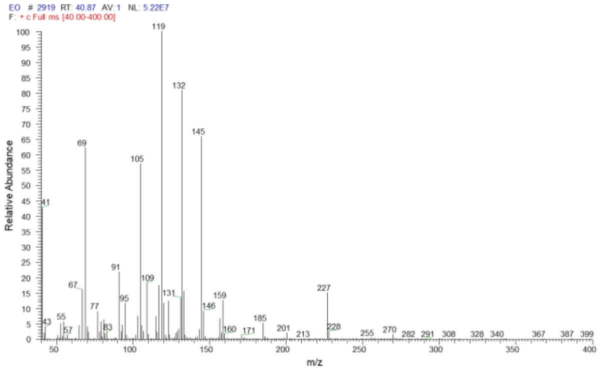

(**d**)

Figure S3. Fragment ions and relative abundance (*m*/*z*, %) of some characteristic volatile compounds identified in *S. sipylea* non-polar extracts. (**a**) β-Caryophyllene (% score of similarity: 35.05). tR: 26.1 min, EI-MS *m*/*z* (%): 41 (85), 53 (23), 55 (28), 67 (42), 69 (100), 77 (36), 79 (59), 81 (28), 91 (63), 93 (71), 105 (58), 106 (29), 107 (40), 119 (28), 120 (25), 133 (64), 147 (20), 161 (21), 204 (M^+, 3). (**b**) Spathulenol (% score of similarity: 48.44). tR: 31.1 min, EI-MS *m*/*z* (%): 41 (64), 43 (100), 55 (22), 67 (33), 69 (44), 71 (29), 77 (30), 79 (38), 91 (55), 93 (41), 105 (58), 106 (25), 107 (38), 119 (48), 131 (26), 145 (21), 147 (24), 159 (34), 162 (22), 205 (38), 220 (M^+, 2). (**c**) Caryophyllene oxide (% score of similarity: 35.01). tR: 31.2 min, EI-MS *m*/*z* (%): 41 (100), 43 (98), 53 (26), 55 (40), 67 (57), 69 (90), 71 (28), 77 (45), 79 (82), 81 (38), 91 (65), 93 (68), 95 (49), 96 (30), 105 (53), 106 (42), 107 (49), 109 (45), 110 (23), 121 (26), 220 (M^+, 1). (**d**) Geranyl-*p*-cymene (% score of similarity: 92.68). tR: 40.9 min, EI-MS *m*/*z* (%): 41 (43), 67 (16), 69 (62), 91 (22), 95 (12), 105 (57), 109 (18), 117 (18), 119 (100), 120 (12), 123 (12), 131 (13), 132 (81), 133 (16), 145 (66), 159 (13), 227 ($[M-CH_3]^+$, 15).
